# Protocol for the modeling the epidemiologic transition study: a longitudinal observational study of energy balance and change in body weight, diabetes and cardiovascular disease risk

**DOI:** 10.1186/1471-2458-11-927

**Published:** 2011-12-14

**Authors:** Amy Luke, Pascal Bovet, Terrence E Forrester, Estelle V Lambert, Jacob Plange-Rhule, Dale A Schoeller, Lara R Dugas, Ramon A Durazo-Arvizu, David Shoham, Richard S Cooper, Soren Brage, Ulf Ekelund, Nelia P Steyn

**Affiliations:** 1 Stritch School of Medicine, Loyola University Chicago, Maywood, IL, USA; 2 Institute of Social & Preventive Medicine, Lausanne University Hospital, Lausanne, Switzerland & Ministry of Health, Republic of Seychelles; 3 Tropical Medicine Research Institute, University of the West Indies, Mona, Kingston, Jamaica; 4 Research Unit for Exercise Science and Sports Medicine, University of Cape Town, Cape Town, South Africa; 5 Kwame Nkrumah University of Science and Technology, Kumasi, Ghana; 6 University of Wisconsin, Madison, WI, USA; 7 MRC Epidemiology Unit, Addenbrooke's Hospital, Cambridge, UK; 8 Health Sciences Research Council, Cape Town, South Africa

## Abstract

**Background:**

The prevalence of obesity has increased in societies of all socio-cultural backgrounds. To date, guidelines set forward to prevent obesity have universally emphasized optimal levels of physical activity. However there are few empirical data to support the assertion that low levels of energy expenditure in activity is a causal factor in the current obesity epidemic are very limited.

**Methods/Design:**

The Modeling the Epidemiologic Transition Study (METS) is a cohort study designed to assess the association between physical activity levels and relative weight, weight gain and diabetes and cardiovascular disease risk in five population-based samples at different stages of economic development. Twenty-five hundred young adults, ages 25-45, were enrolled in the study; 500 from sites in Ghana, South Africa, Seychelles, Jamaica and the United States. At baseline, physical activity levels were assessed using accelerometry and a questionnaire in all participants and by doubly labeled water in a subsample of 75 per site. We assessed dietary intake using two separate 24-hour recalls, body composition using bioelectrical impedance analysis, and health history, social and economic indicators by questionnaire. Blood pressure was measured and blood samples collected for measurement of lipids, glucose, insulin and adipokines. Full examination including physical activity using accelerometry, anthropometric data and fasting glucose will take place at 12 and 24 months. The distribution of the main variables and the associations between physical activity, independent of energy intake, glucose metabolism and anthropometric measures will be assessed using cross-section and longitudinal analysis within and between sites.

**Discussion:**

METS will provide insight on the relative contribution of physical activity and diet to excess weight, age-related weight gain and incident glucose impairment in five populations' samples of young adults at different stages of economic development. These data should be useful for the development of empirically-based public health policy aimed at the prevention of obesity and associated chronic diseases.

## Background

Populations all over the world are experiencing rapid increases in the prevalence of obesity and diabetes [1-5]. To date, the public health response to the emerging epidemics has been largely ineffective. As a first line of response, professional bodies and government organizations have issued prevention guidelines, all of which include recommendations on levels of physical activity (PA) required to prevent weight gain [6-10]. There is limited direct evidence, however, that can be brought to bear on the question of whether the obesity epidemic has resulted primarily or even partially from population-wide declines in habitual PA [11]. Thus, even if fully implemented, it is not clear that the current recommendations on PA would impact the trend in age-related weight gain.

Energy balance is defined by the direct relationship between energy intake and expenditure. Fat mass accumulation and subsequently obesity can only result from an excess of calories consumed over calories expended. It is often assumed, therefore, that a similarly straightforward, simple relationship exists between variation in PA within the range of normal for a population and the risk of weight gain. The second hypothesis which follows from this line of reasoning is that population-level weight gains taking place have resulted, at least in part, from declining PA, and that small increases in PA for individuals will prevent weight gain. In fact, neither of these lines of thoughts is justified by theory alone or supported by evidence. Volitional increases in physical expenditure are typically accompanied by increases in energy intake [12,13], while excess intake can be stimulated independently by high caloric density of food, changes in food availability, and eating patterns. As pointed out repeatedly by investigators in this field [14-17], the regulation of energy stores and body composition must be seen as a complex, dynamic process influenced by the interplay between factors that modify both intake and expenditure and these proximal factors are admittedly importantly influenced by distal environmental factors at the society and global levels [11]. An individual ultimately responds to biological stimuli, e.g., the hormones that control satiety; and environmental cues, e.g. food aromas or congested walkways, to modulate energy balance [18-21]. Whether variation in patterns of activity observed in modern, free-living populations plays a key role in this process is thus largely unknown.

Lifestyle trends are clearly taking place in many societies; however, it is difficult to identify clear temporal associations with increases risk of obesity. As demonstrated in the trends from the CDC's Behavioral Risk Factor Surveillance System and the National Health and Nutrition Examination Surveys conducted since the 1960's, the US experienced an upward deviation of the trend for body mass index (BMI) in the mid-1980s, resulting in the current "obesity epidemic" [22]. However, there was no obvious temporally related break in activity patterns or eating habits [11,22]. In the absence of direct evidence most observers have defaulted to a "common sense" explanation that people are "eating more and exercising less". Unfortunately, this formulation is both an oversimplified concept and unsubstantiated causally [11] and there have been relatively few longitudinal studies investigating this premise. Thus the central purpose of our project is to test whether change in PA can be identified as a contributory mechanism to the population-wide weight gain, and, if so, to quantify its importance.

It is repeatedly stated that the environment of modern society is "obesogenic" in part because of the greatly reduced need for physical exertion and increased supply of foods universally [23-25]. While it seems self-evident that industrialized societies require less PA across a range of domains, the empirical data on this question lead to the opposite conclusion. As summarized by Ferro-Luzzi and Martino [26], "*The seemingly obvious conclusion that energy expenditure is systematically higher in Third World countries is not supported by the evidence... (Based on a review of the data) we conclude, therefore, that there are no systematic differences in the level of habitual activity between developed and developing countries" *[26]. Additionally, the results of a recent meta-analysis of total energy expenditure and PA levels in adult samples found no difference in either, after adjustment for body size, between developing and more industrialized countries [27]. While PA has many indisputable health benefits, its role in the regulation of body weight requires careful additional study particularly in populations in early stages of the epidemiologic transition. We recognize that surveillance for PA will be crucial to unravel the causes of the obesity epidemic, and it must be conducted using objective measures.

### Modeling the Epidemiologic Transition Study Hypotheses and Aims

The Modeling the Epidemiologic Transition Study (METS) was designed to test four hypotheses associated with the relationship between PA, body weight and diabetes and cardiovascular disease (CVD) risk. We hypothesized that: 1) population mean levels of PA are negatively related to population mean levels of obesity and relative weight, 2) PA is negatively related to percent body fat in the study populations at baseline, independent of dietary intake, 3) PA is negatively related to change in body weight during follow-up, independent of dietary intake, and as an exploratory hypothesis, 4) PA modifies the association between adipocytokines (e.g., adiponectin and leptin) and hormones (e.g., ghrelin) and weight regulation and insulin sensitivity.

In order to test these hypotheses, the investigators of METS have enrolled 500 participants, ages 25-45 years, from each of five African-origin populations, i.e., Ghana, South Africa, Seychelles, Jamaica and the United States. In all participants at baseline PA was measured using accelerometry and dietary intake by 24-hour dietary recalls, fasting glucose/insulin, and adipocytokines were measured by standard laboratory methods, and social and environmental factors, PA patterns and alcohol, smoking and medication histories were assessed by questionnaire. In a random subset of 75 participants from each site at baseline, total energy expenditure (TEE) was measured using the doubly labeled water (DLW) method and resting energy expenditure (REE) by indirect calorimetry. Follow-up measurements of weight, waist circumference and blood pressure will be made at 12-months and all measures except dietary intake, TEE and REE will be repeated at 24-months. The statistical analyses will examine the relationship between PA and body composition within and among populations at baseline and between PA and weight change at follow-up, and assess inter-relations of PA, diet, glucose, insulin and adipocytokines.

## Methods and Design

### Design and Settings

Twenty-five hundred adults, ages 25-45, were enrolled in METS between January 2010 and September 2011 and have had energy expenditure, dietary intake, body weight and composition, and biomarkers of obesity and diabetes measured at baseline. The participants will all be followed over the subsequent 24 months to assess change in body weight, composition, and diabetes and CVD risk. Five hundred participants, 50% of whom are female, were enrolled in each of five study sites: rural Ghana, urban South Africa, Seychelles, urban Jamaica and suburban United States (Chicago area). The populations sampled are of African descent and provide a range of body sizes, i.e., the mean BMIs of adults from the study sites vary from a low of about 24 kg/m^2 ^in rural Nkwantakese (Ghana) to a high of 31 kg/m^2 ^in suburban Maywood (USA). The study sites also represent a range of social and economic development as defined by the UN Human Development Index (HDI) 2010: Ghana is defined as a low HDI country, South Africa as middle HDI, Jamaica and Seychelles as high HDI and the US as a very high HDI country [28].

The in-country study sites include the town of Nkwantakese in the Afigya-Kwabre District of the Ashanti Region of Ghana and its surrounding villages. The town is situated to the southwest of Agona Ashanti the District Capital, and is about 20km from Kumasi with a population of approximately 17,000. Khayelitsha is the 3^rd ^largest township in South Africa and is adjacent to the city of Cape Town. The population is about 500,000 with 80 percent of the residents living in temporary housing and 40 percent unemployed. The study site in Jamaica is in Kingston, the capital and largest city with a population of 651,880. In Seychelles, individuals have been recruited from the main island of the archipelago, Mahé, which includes approximately 75,000 inhabitants for a surface of 155 km^2^. Mahé can be qualified as semi-urban and its economy is mainly driven by tourism, industrial fishing and services. Seychelles is located approximately 1,600 km east of Kenya in the Indian Ocean, and approximately 2,000 km north of the island of Mauritius, and has a total population of about 87,000. Maywood, in the US, is an African-American working class community adjacent to the western border of Chicago, Illinois, with a population of approximately 24,903 people. The research clinic is on the campus of Stritch School of Medicine, Loyola University, within walking distance of most neighborhoods and is well known to local residents. The coordinating center for METS is also located in Maywood, IL at Loyola University.

We excluded individuals with obvious infectious diseases (including active malaria), pregnant or lactating women, and HIV positive individuals. Any individual that has a condition preventing them from engaging in normal physical activities, such as severe osteo'- or rheumatoid arthritis, lower extremity disability, was also excluded from METS. Population-based surveys have been previously carried out in each of the sites, thus investigators from each site decided upon the best means of recruiting a representative sample from their respective communities. In Nkwantakese, Ghana, a simple random sample was generated for the age-range of the study from the population census for Nkwantakese and its surrounding villages. In both Seychelles and South Africa sex- and age-stratified random samples were generated from their respective national censuses. In Kingston, Jamaica, districts were randomly sampled; beginning from a fixed point in each district (e.g., the north-west corner), and door-to-door recruitment took place. Similarly, in Maywood, IL, USA, all city blocks in the community were randomized and door-to-door recruitment was conducted.

### Ethics Approval

The protocol for METS was approved by the Institutional Review Board of Loyola University Chicago, IL, USA; the Committee on Human Research Publication and Ethics of Kwame Nkrumah University of Science and Technology, Kumasi, Ghana; the Research Ethics Committee of the University of Cape Town, South Africa; the Board for Ethics and Clinical Research of the University of Lausanne, Switzerland; the Ethics Committee of the University of the West Indies, Kingston, Jamaica; and the Health Sciences Institutional Review Board of the University of Wisconsin, Madison, WI, USA. Written informed consent was obtained from all participants.

### Measurements

Baseline, 12-month and 24-month follow-up examinations will be conducted for the METS study. The project coordinators for each field site were jointly trained and certified in all measurement protocols by coordinating center staff; the measurements included in METS are presented in Table 1. All measurements were undertaken at outpatient clinics located in each of the METS communities.

**Table 1:** Study Measures

	Baseline	12-Mo	24-Mo
**Objectively Measured Energy Expenditure**			
Physical activity (Actical)	X		X
Total energy expenditure (DLW;subset N = 375)	X		
Resting energy expenditure (indirect calorimetry; subset N = 375)	X		
**Self-Reported Measures**			
Physical Activity (GPAQ)	X		X
Dietary Intake (24-hr Recall)	X		
Medication & supplement use	X	X	X
Smoking status & alcohol consumption	X	X	X
Health history	X	X	X
Household SES, education level, industry & occupation	X		X
**Body Composition**			
Bioelectrical impedance analysis	X		X
Isotope Dilution (subset N = 375)	X		X
**Clinical Measures**			
Weight, height, waist & hip circumferences, blood pressure & pulse	X	X	X
**Biochemical Measures**			
Hba1c, total cholesterol, HDL and LDL cholesterol, triglyceride, glucose, insulin, adiponectin, leptin, ghrelin, urinary albumin & creatinine, and T_4_, T_3 _& TSH in subset (N = 375)	X		

#### Physical Activity (Accelerometer)

Physical activity was assessed using the Actical accelerometer (Phillips Respironics, Bend, OR, USA) in all 2500 METS participants. Previous studies have shown that accelerometer-based activity monitors can discriminate differing intensities of activity [29-34], making it possible to adequately characterize each of the study communities with regard to overall and subcomponents of PA. The activity monitor records the intensity, duration and frequency of physical motion through the use of an accelerometer which produces a variable electrical current based on the combination of the amplitude and frequency of motion. Accelerometers are omnidirectional motion sensors that count the vertical and horizontal acceleration of the user. This information is stored within the instrument as activity counts per epoch, or specified subunit of time, e.g., per minute. As the intensity of the activity increases, so does the number of activity counts per epoch.

The monitor was worn at the waist, positioned just behind the left hip. Each participant was asked to wear the activity monitor at all times over 8 days, including during sleep; the only time the monitor should be removed was while bathing, showering, or swimming. We chose to monitor all participants for six complete days (i.e., a total of 8 days after taking the two partial days into consideration); based on preliminary work, this will provide a good level of reliability at 0.83-0.92%.

#### Total Energy Expenditure (Doubly Labeled Water Method)

A subset of 75 participants per site were enrolled in the DLW protocol (N = 375 total). For these participants, PA energy expenditure was calculated as the difference between TEE as measured using DLW and REE (PA = TEE - REE - TEF; where TEF, thermic effect of food, will be estimated as 10% of TEE) [35].

Detailed descriptions of the DLW method and laboratory analysis have previously been published [36-39]. In brief, TEE was measured over a 7-day period. On the morning of the initial clinic examination, prior to the measurement of REE, a spot urine collection was made and the participant given an oral dose of DLW containing approximately 1.8 g of 10% H_2_^18^O and 0.12 g 99.9% ^2^H_2_O per kg body water. Spot urine samples were then collected at approximately 1, 3 and 4 hours after isotope administration. Participants collected an early morning urine void on day 7 and returned to the clinic later the same day (± 1 day) to provide a final urine sample. All urine samples were aliquotted in duplicate and stored in 5-mL o-ring-sealed cryovials at -20°C. Mass spectrometric analyses of the DLW urine samples were carried out at the Stable Isotope Core Laboratory at University of Wisconsin, Madison, WI, USA. The CO_2 _production was calculated using equation 6.6 of the IAEA technical [40] and energy expenditure using the modified Weir equation [41]. The country specific average RER from dietary records was used for the latter.

#### Resting Energy Expenditure

Resting EE was measured in the DLW subsample using indirect calorimetry. In the US, Ghana and Seychelles sites, REE was measured using the MaxIIa indirect calorimeter (AEI Technologies, Aurora, IL, USA); in Jamaica the Columbia Instruments Oxymax 4.0. (Columbus, Ohio, USA) was used; and in South Africa, the VMax indirect calorimeter by SensorMedics (Viasys Health Care, Waukegan, IL, USA) was used. Cross-validation of instruments was carried out through external calibrations. The investigators have had extensive experience in the measurement of REE across multiple sites, with over 2500 measurements made in the US and abroad [42,43].

The detailed description of measurement of REE using indirect calorimetry has been previously published [42-45]. Participants were asked to fast from 10 pm the evening prior to the initial examination and were rested for at least 15 minutes prior to the REE measurement. Respiratory gases were collected for 30 minutes, the first 10 minutes of data were discarded and last 20 minutes used to estimate REE. Oxygen and carbon dioxide were continuously sampled during the procedure and minute-by-minute consumption and production values were calculated; EE was calculated according to the modified Weir equation [41].

#### Physical Activity by Questionnaire

All participants had PA also assessed using the Global Physical Activity Questionnaire (GPAQ, version 2) [46]. The GPAQ was developed by the World Health Organization (WHO) as part of the WHO STEPwise approach to chronic disease risk-factor surveillance [47] to produce reliable and valid estimates of PA for use in developing countries. The main outcome variables are: a categorical variable of total PA (high, moderate and low) and a continuous variable of PA within the domains of work, transport and leisure.

#### Dietary Intake

Each participant has completed two 24-hour recalls using the multiple pass method [48-50], one at the initial baseline examination and the second when the activity monitor is collected, typically 6-9 days later. At each site, the 24-hour recalls were collected by centrally trained interviewers using pen and paper, scanned and sent via secure FTP [51] to the Coordinating Center at Loyola University Chicago where data entry and analysis using the Nutrient Data System for Research (NDSR; University of Minneapolis, MN, USA) [48-50] took place. Food ingredient identification and portion size estimation were augmented using photographs and usual portions of local foods assembled at each site prior to study initiation; this methodology is based on the Dietary Assessment Education Kit developed by one of the study consultants for the Medical Research Council South Africa [52]. Primary endpoints of interest are total energy intake and macronutrient composition (i.e., % kcals from fat, carbohydrate and protein), as well as indicators of intake of processed foods (e.g., # of sweetened beverages, pre-packaged foods and restaurant fast foods per day) and of intake of fruits, vegetables, as well as site-specific commonly eaten foods. While we recognize the importance of micronutrients for overall health and well-being of individuals, many nutrient databases lack sufficient data on local food micronutrient content.

#### Smoking Status & Alcohol Consumption

Participants were classified as non-smoker, occasional or smoker based on questions on cigarettes, cigar or pipe smoking and chewing tobacco use. In addition, there were multiple questions concerning alcohol use in an effort to describe type, volume and frequency of consumption.

#### Health History, Medication & Supplement Use

Basic health history information, with a focus on cardiovascular conditions and diabetes, was collected including age of first diagnosis where applicable. Participants were asked about medication and dietary supplement use, with emphasis on vitamin D and calcium supplements.

#### Household SES, Education and Occupation

Fifty-four questions were included which covered general household characteristics, participant and significant other's occupation, parental education and household assets and amenities. These questions were based on the Core Welfare Indicators Questionnaire from the World Bank, designed to monitor social indicators in Africa [53].

#### Body Composition (Bioelectrical Impedance Analysis)

Body composition was assessed in all participants using bioelectrical impedance analysis (BIA); BIA measures the impedance to the flow of an applied mild alternating current by body tissues. The measured impedance of body tissues can be used to estimate total body water, from which fat-free mass and fat mass can be calculated [54]. With participants in the supine position with limbs abducted, current-supplying electrodes were placed on the dorsal surfaces of the right hand and foot at the metacarpals and metatarsals, respectively, and detection electrodes were placed at the pisiform prominence of the right wrist and the anterior surface of the true ankle joint [54]. A single-frequency instrument (BIA Quantum, RJL Systems, Clinton Township, MI) was attached to the electrodes and generated an excitation current of 800 μA at 50 kHz; resistance and reactance measures were recorded. Unless data from the isotope dilution analyses, described below, indicate a need for the development of separate equations for our sample, we propose to use pre-existing validated BIA equations [55] for estimation of total body water. Impedance measurements will be taken at baseline and at the 24-month examination to estimate change in body composition.

#### Body Composition (Isotope Dilution)

Total body water was measured using isotope dilution in the DLW subset of participants at baseline and again at the 24-month examination. The basis of this measurement is the dilution principle: total body water was calculated using the measurement of the abundance of either isotope (deuterium or 18-oxygen) from the DLW procedure after complete equilibration with body water [56] and correction for non-aqueous exchange of 1.042 and 1.007, respectively [40]. Fat-free mass was calculated using a hydration constant (0.732 [57]) from total body water, and fat was calculated as the difference between body weight and fat-free mass [58]. The 375 isotope dilution measurements at both baseline and follow-up will be used to calibrate BIA measurements for change in individual body composition.

#### Height, Weight & Circumferences

At the initial clinic examination, height, weight, and waist and hip circumferences were measured. Weight was measured without shoes with the participant dressed in light clothing to the nearest 0.1 kg using the same model standard calibrated balance at all 5 sites (Seca 770, Hamburg, Germany). Height was measured to the nearest 0.1 cm using a stadiometer (e.g. Invicta Stadiometer, Invicta, London, UK) without shoes and with the participant's head held in the Frankfort plane. Waist circumference was measured to the nearest 0.1 cm at the umbilicus using a flexible metal tape measure (Gulick, Creative Engineering, Michigan, USA). Hip circumference was measured to the nearest 0.1 cm at the point of maximum extension of the buttocks. For both circumferences, repeat measures will be taken and if the two measurements differ by more than 0.5 cm, a third measurement was taken.

#### Blood Pressure

Blood pressure was measured using the protocol and training procedures developed for our ongoing international hypertension studies [44,59-62]. Systolic and diastolic blood pressure and pulse were measured using the Omron Automatic Digital Blood Pressure Monitor (model HEM-747Ic, Omron Healthcare, Bannockburn, IL, USA). With the antecubital fossa at heart level, three measurements were made at each of two time points separated by approximately 60 minutes.

#### Biochemical Measures

Participants were asked to fast from the evening prior to the baseline clinic examination. Fasting blood samples were drawn for analysis of adipose-related hormones and adipocytokines, glucose, insulin, lipids, albumin. A random spot urine sample was collected for assessment of urinary creatinine and albumin. The blood samples were processed and plasma or serum separated within two hours of collection and stored at -80°C in the laboratory at each study site. Fasting plasma glucose was measured using the glucose oxidase method at each site at the time of collection. Insulin, total ghrelin, leptin and adiponectin from all sites were measured using radioimmunoassay kits at the departmental laboratory at Loyola University Chicago (Linco Research, Inc., St. Charles, MO). All remaining assays were conducted at the Zentrum fϋr Lambormedizin, Leiter Klinische Chemie und Hämatologie, St. Gallen, Switzerland.

### Participant Safety

All protocols for METS were approved by the Institutional Review Board or Ethics Committee of all participating institutions. Written informed consent was obtained from all participants in all study sites; all participants received a copy of the signed consent containing site-specific contact information in case of questions or complaints.

Participants were provided results of plasma glucose levels and blood pressure at the time of their baseline or follow-up clinic examinations. In cases where elevated plasma glucose or blood pressure was identified, according to clinical cut-points, participants were referred to appropriate clinics or their own physicians.

### Data Management

Data management is centralized at the coordinating center at Loyola University Chicago. All data forms, questionnaires and dietary recall instruments are scanned and, along with electronic Actical data files, are sent via secure FTP (Bitvise Tunnelier [51]) to the data manager at the coordinating center. All scanned forms are coded by experienced, trained personnel and double data entry is carried out. A series of logic checks are then performed and, when outliers are encountered, discrepancies are followed up with staff at the appropriate field site.

### Statistical Analysis

#### Analytical Plan

In the analysis phase of METS descriptive characteristics from each study site (e.g., mean levels and distributions of PA, dietary factors, body size and composition, adipocytokines and hormones, and prevalence of hyperglycemia and high blood pressure) will be explored and the univariate correlation structure for the continuous variables will be described. Cross-sectional, multivariable regression models will then be constructed to assess the role of PA as a predictor of weight and risk factor status, independent of diet. Similar analyses will be conducted to assess the relationship between PA and change in weight and risk factors. An important focus of these analyses will be looking for potential heterogeneity of risk relationships across sites. Three main aspects are of interest, namely whether the PA-risk factor (e.g. insulin level) relationship is present, or of the same magnitude, in each sample and whether there exists co-variation with the mean level of PA; testing for interaction will be undertaken where appropriate.

The magnitude of association between PA and risk factors will be assessed using both regression coefficients and standardized regression coefficients, allowing within and between site comparisons. For situations in which the standard deviation of the variables differ across sites standardized regression coefficients will be obtained using pooled estimates of the mean and standard deviation. Multilevel models will then be applied, in which the site specific variable, mean PA, will be used to estimate the effect of the average PA on the regression coefficient. Multiplicative interaction terms will be included in regression models to assess how much site modifies the association between PA and the risk factor.

There will be three weight determinations over time: one at baseline, a second after 12 months, and a final assessment 2 years after entering the study. Mixed effect models will be used to assess the association between PA and weight change. This approach will allow for the use of all the available information thus improving the statistical power [63], and account for the within-subject correlation of the weight determinations, as well as adjust for subject-specific covariates that may confound the associations. In addition, the dependence of weight change on PA values can be modeled by the use of random coefficients. Potential confounders will include demographic variables and energy intake variables (e.g. total intake, percent calories from fat, etc). Similar multilevel analysis will be used to assess the associations between PA and change in risk factors such as blood pressure and fasting glucose concentration.

#### Sample Size Estimation

A statistical justification for the selected sample size is provided for each aim of the study. Cross-sectional association of PA to obesity/relative weight across sites: Through preliminary studies, we have estimated that the within-site correlation between PA and adiposity of individual data could be as large as 0.6, in addition we anticipate that the correlation between mean PA levels and mean body composition will exceed 0.85. A one-sided Fisher's Z-transformation 5%-significance test will have over 55% power to detect this correlation with 5 values corresponding to the 5 sites of the study. However, after dividing our samples by gender there will be 10 observations (2 × 5 countries), resulting in over 95% statistical power. Assuming an effective sample size of 7, to account for within-country correlations, the statistical power is 80%. Note that additional power will be gained when weighted linear regression methods are applied to estimate the correlation between PA and adiposity using meta-regression.

Cross-sectional association of PA to adiposity and CVD risk factors within site: The correlation between PA as measured using DLW and body composition is between -0.6 and -0.5. A correlation of -0.33 or smaller will be detectable with 83% power and maximum probability of type I error alpha = 5% using a one-sided Fisher's Z test and 75 subjects per site [64].

Longitudinal association of PA to change in body weight within site: PA, measured using accelerometry, is associated with changes in body composition. Two PA groups will be formed by using the median total PA and moderate-vigorous PA as cut points. Mean changes in weight between groups will be compared in a longitudinal analysis, adjusting for baseline values [65]. In this method the following assumptions are made: the correlation between any 2 weight determinations is ~ 0.60 and the minimum acceptable difference between the means is 0.25 standard deviations (i.e., effect size equals 0.25) [66]. There will be one baseline measure of weight and two follow-up measurements. An analysis of covariance approach will be used to adjust for baseline values. To detect the pre-specified minimum difference with 90% statistical power we needed to accrue 460 subjects per PA group (230 in the low and 230 in the high group).

## Discussion

Although the "obesity epidemic" has received enormous attention in recent years, the manner and mechanisms through which the energy budget is being perturbed are still not well understood. In particular, the role of declining PA as a causal mechanism in population-wide increases in relative weight deserves much greater attention and new methods employed in epidemiologic research are required before we can base causal inferences or public health recommendations on something more than assumptions. As with other diseases, international comparisons may be particularly informative. METS will provide information on the relative contributions of physical activity and energy intake to excess relative weight, weight gain and diabetes risk in young adults from five African-origin populations at different stages of economic development.

The tests of our study hypotheses will be "two-sided". That is to say, the presence or absence of an association can be given a clear interpretation. Of course, given the body of evidence that increased PA has health advantages, absence of an association would not be taken to suggest that PA is of no value. Rather, the correct interpretation would be that declining PA is not likely to be the driving force behind the rise in obesity, nor will modest increases in PA be adequate to reverse the trends. These data will be critically important for the development of meaningful public health policy targeted at the prevention of obesity, particularly for populations at early stages of the epidemiologic transition.

## Competing interests

The authors declare that they have no competing interests.

## Authors' contributions

AL is the overall principal investigator for METS. PB, TEF, EVL, and JPR are the principal investigators at each of the four study sites outside of the United States. DAS is responsible for the doubly labeled water analyses and data interpretation. LRD is the project coordinator. RADA is the study statistician. DS is the social epidemiologist and RSC is the cardiovascular epidemiologist. SB and UE are consultants aiding in the analysis and interpretation of accelerometry data and NPS is a consultant assisting with the dietary data. AL, LRD and RADA drafted the manuscript. All authors approved the final manuscript.

## List of abbreviations

PA: Physical activity; CDC: Centers for Disease Control and Prevention; BMI: Body Mass Index; METS: Modeling the Epidemiologic Transition Study; CVD: Cardiovascular disease; TEE: Total energy expenditure; DLW: Doubly labeled water; REE: Resting energy expenditure; USA: United States of America; UN: United Nations; HDI: Human development index; TEF: Thermic effect of food; RER: Respiratory exchange ratio; GPAQ: Global physical activity questionnaire; WHO: World Health Organization; FTP: File transfer program; NDSR: Nutrient data system for research; BIA: Bioelectrical impedance analysis.

**Figure 1:**
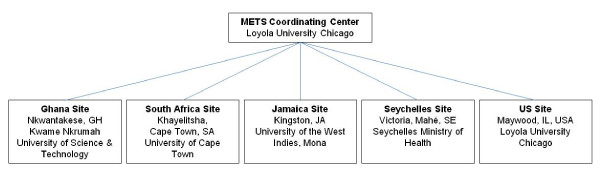
**Study sites and affiliated institutions for the Modeling the Epidemiologic Transition Study (METS)**.

## Pre-publication history

The pre-publication history for this paper can be accessed here:

http://www.biomedcentral.com/1471-2458/11/927/prepub
